# Bacterial, Viral and Parasitic Pathogens and Colorectal Cancer

**DOI:** 10.3390/cancers15133353

**Published:** 2023-06-26

**Authors:** Ikuko Kato

**Affiliations:** Department of Oncology and Pathology, Wayne State University School of Medicine, Detroit, MI 48201, USA; katoi@karmanos.org

Several viral, bacterial, and parasitic pathogens have been designated as human carcinogens by the World Health Organization [1,2]. In fact, infection has been estimated to account for 15% of cancer incidence worldwide [1] and represents one of the important modifiable risk factor for cancer. Yet, despite the fact that the gut contains trillions of microorganisms [3], to date no specific microbial pathogens have been conclusively linked to colorectal cancer (CRC), the type of cancer with the second highest rates of cancer mortality in the United States [4]. However, growing evidence suggests that dysbiosis, imbalances in gut resident bacterial populations, can promote carcinogenesis in humans through the induction of chronic inflammation, immune-subversion, and production of carcinogenic metabolites [5,6,7,8]. In addition, the potential involvement of various pathogens, including Human Papillomavirus, polyomavirus, cytomegarovirus, *Helicobacter pylori*, *Fusobacterium nucleatum*, *Bacteroides fragilis*, *Streptococcus gallolyticus*, *Escherichia coli*, *Schistosoma japonicum*, and *Blastocystis*, has long been recognized [9,10,11,12,13]. Given an increasing number of publications in this field, an update and synthesis of the current knowledge is warranted.

To this Special Issue of *Cancers*, four groups of investigators contribute their work to advance our current knowledge concerning the potential roles of bacterial and parasitic pathogens as causative factors and treatment targets for CRC. Redondo et al. report the association between colon cancer and microsporidia, obligate intracellular parasites which cause severe infections in immunosuppressed subjects due to diarrheal disease and disseminated infection [14]. The study focused on *Encephalitozoon* species, analyzing colorectal mucosa and sera from 87 cases and 25 controls. *Encephalitozoon* in colon tissue was visualized with an immunofluorescence antibody test. The authors found that *Encephalitozoon* DNA was only detectable in colon tissues from cancer cases (30% vs. 0%) and IgG and IgE antibody titers were statistically significantly higher in colon cancer cases than in controls. Their findings warrant further investigation into these parasites given the reported increases in mutation frequency induced by this organism.

Wang and Fu synthesized the knowledge concerning three genotoxins produced by *Escherichia coli*, specifically cytolethal distending toxin (CDT), colibactin encoded by the *pks* pathogenicity island of group B2 strains, and UshA, which acts as a type III secretion system effector, for their key roles in CRC initiation and promotion [15]. The authors clarified similarities and differences of these genotoxins. While all cause double-strand DNA breaks and CDT and UshA single strand DNA breaks, each have their own characteristics, i.e., nicking or relaxation activity by CDT, alkylation of chromatin DNA by colibactin, and direct digestion of DNA substrates by UshA. The authors further point out the scenario that surviving host cells from genotoxin injection accumulate genomic instability and thus acquire malignant traits.

Löwenmark et al. analyzed survival data of 257 CRC patients according to abundances of two common oral pathogens, *Parvimonas micra* and *Fusobacterium nucleatum* in feces and tumor tissue, which were quantified by qPCR [16]. While they did not find differences in survival by fecal levels of these two bacterial species, they did find decreased five-year survival associated with higher abundance of these bacteria in tumor tissue. Furthermore, they revealed significant associations of higher abundance of *P. micra* and *F. nucleatum* with tumor molecular characteristics, i.e., *BRAF^V600E^* mutation, and the microsatellite instability subtype. It was also found that *P. micra* and *F. nucleatum* often colonized the same tumors, suggesting potential synergistic interactions within biofilm. While the finding on prognostic effects of *P. micra* is relatively novel compared with those of *F. nucleatum*, combined and independent effects of these two bacteria were not analyzed within this paper and the temporal relationship of accumulation of these two bacteria was not clear. Thus, further investigations on interactions among these and other biofilm forming bacteria, as well as specific oncogenic pathways activated by *P. micra* are needed to determine their prognostic values.

Because of a growing number of recent publications linking *Fusobacterium nucleatum* to CRC, Altruki et al. developed the computational pipeline that will help in the identification of natural therapeutic products against the microbial targets [17]. They performed the high throughput screening of core genomes of 14 *F. nucleatum* strains related to CRC through a pan-genome integrated subtractive genomics approach and identified 12 loci as drug targets. Subsequently, riboflavin synthase was selected as a therapeutic target and virtual screening led to the identification of three natural compounds as potential inhibitors. Dynamics simulation analysis revealed the stability of these compounds within the binding pocket and the virtual absorption, distribution, metabolism, excretion, and toxicity profiling confirmed their safety. The authors further suggest that use of artificial intelligence would improve the performance of these tools. These bioinformatics approaches are likely to serve as the preliminary step towards inhibition of oncogenic microbiome that is enriched in CRC and of which causal association with CRC is confirmed. It is interesting to see if efficacy of any of these three compounds will be confirmed in animal models and move forward to clinical trials.

Despite the small collection, this Special Issue covered a range of topics in microbial pathogens and CRC, from cancer initiation, progression, and therapeutic target to prognostic markers. While the association of *Encephalitozoon* and *P. mirca* with CRC [14] and its molecular subtype [16] as well as the prognostic effect of *P. mirca* are both intriguing, they were based on small numbers of samples, and thus independent replication/validation is required. The increased presence of these microorganisms in tumor tissues per se does not support the causative role or cancer promoting activities; they may simply be a marker of dysbiosis or consequences of neoplastic change of colorectal mucosa such as mucin depletion [18,19]. Nevertheless, it is possible that they may serve as biomarkers for early detection or risk stratification factors in treatment choices. In addition to individual pathogens or taxonomic units, studies on bacterial genotoxins (as reviewed by Wang and Fu [15]) as well as other exotoxins produced by multiple bacterial species in the gut [20] and their genetic loci that encode those toxins are crucial in elucidating oncogenic pathways and molecular therapeutic targets of CRC induced by intestinal bacteria. In addition, research aimed to identify exogenous, endogenous, and other microbial factors (including bacteriophages) that foster aquation, colonization, and persistence of potentially oncogenic microorganisms are important to facilitate CRC prevention.

## References

[1] Plummer M., de Martel C., Vignat J., Ferlay J., Bray F., Franceschi S. (2016). Global burden of cancers attributable to infections in 2012: A synthetic analysis. Lancet Glob. Health.

[2] van Elsland D., Neefjes J. (2018). Bacterial infections and cancer. EMBO Rep..

[3] Schmidt T.S., Hayward M.R., Coelho L.P., Li S.S., Costea P.I., Voigt A.Y., Wirbel J., Maistrenko O.M., Alves R.J., Bergsten E. (2019). Extensive transmission of microbes along the gastrointestinal tract. Elife.

[4] Siegel R.L., Miller K.D., Fuchs H.E., Jemal A. (2022). Cancer statistics, 2022. CA A Cancer J. Clin..

[5] Joossens M., Huys G., Cnockaert M., De Preter V., Verbeke K., Rutgeerts P., Vandamme P., Vermeire S. (2011). Dysbiosis of the faecal microbiota in patients with Crohn’s disease and their unaffected relatives. Gut.

[6] Marchesi J.R., Dutilh B.E., Hall N., Peters W.H.M., Roelofs R., Boleij A., Tjalsma H. (2011). Towards the Human Colorectal Cancer Microbiome. PLoS ONE.

[7] Hajishengallis G., Lamont R.J. (2014). Breaking bad: Manipulation of the host response by Porphyromonas gingivalis. Eur. J. Immunol..

[8] Kuper H., Adami H.-O., Trichopoulos D. (2000). Infections as a major preventable cause of human cancer. J. Intern. Med..

[9] Burnett-Hartman A.N., Newcomb P.A., Potter J.D. (2008). Infectious agents and colorectal cancer: A review of Helicobacter pylori, Streptococcus bovis, JC virus, and human papillomavirus. Cancer Epidemiol Biomark. Prev..

[10] Duijster J.W., Franz E., Neefjes J., Mughini-Gras L. (2021). Bacterial and Parasitic Pathogens as Risk Factors for Cancers in the Gastrointestinal Tract: A Review of Current Epidemiological Knowledge. Front. Microbiol..

[11] Hamid H.K.S. (2019). Schistosoma japonicum-Associated Colorectal Cancer: A Review. Am. J. Trop. Med. Hyg..

[12] Taghipour A., Rayatdoost E., Bairami A., Bahadory S., Abdoli A. (2022). Are Blastocystis hominis and *Cryptosporidium* spp. playing a positive role in colorectal cancer risk? A systematic review and meta-analysis. Infect. Agent. Cancer.

[13] Massimino L., Lovisa S., Antonio Lamparelli L., Danese S., Ungaro F. (2021). Gut eukaryotic virome in colorectal carcinogenesis: Is that a trigger?. Comput. Struct. Biotechnol. J..

[14] Redondo F., Hurtado-Marcos C., Izquierdo F., Cuéllar C., Fenoy S., Sáez Y., Magnet Á., Galindo-Regal L., Uribe N., López-Bañeres M. (2022). Latent Microsporidia Infection Prevalence as a Risk Factor in Colon Cancer Patients. Cancers.

[15] Wang Y., Fu K. (2023). Genotoxins: The Mechanistic Links between Escherichia coli and Colorectal Cancer. Cancers.

[16] Löwenmark T., Löfgren-Burström A., Zingmark C., Ljuslinder I., Dahlberg M., Edin S., Palmqvist R. (2022). Tumour Colonisation of Parvimonas micra Is Associated with Decreased Survival in Colorectal Cancer Patients. Cancers.

[17] Alturki N.A., Mashraqi M.M., Jalal K., Khan K., Basharat Z., Alzamami A. (2022). Therapeutic Target Identification and Inhibitor Screening against Riboflavin Synthase of Colorectal Cancer Associated *Fusobacterium nucleatum*. Cancers.

[18] Sakai E., Morioka T., Yamada E., Ohkubo H., Higurashi T., Hosono K., Endo H., Takahashi H., Takamatsu R., Cui C. (2012). Identification of preneoplastic lesions as mucin-depleted foci in patients with sporadic colorectal cancer. Cancer Sci..

[19] Gupta B., Das P., Ghosh S., Manhas J., Sen S., Pal S., Sahni P., Upadhyay A.D., Panda S.K., Gupta S.D. (2017). Identification of High-Risk Aberrant Crypt Foci and Mucin-Depleted Foci in the Human Colon With Study of Colon Cancer Stem Cell Markers. Clin. Colorectal. Cancer.

[20] Silbergleit M., Vasquez A.A., Miller C.J., Sun J., Kato I. (2020). Progress in Molecular Biology and Translational Science. Oral and Intestinal Bacterial Exotoxins: Potential Linked to Carcinogenesis.

